# Editorial: Insights in hypertension: 2022

**DOI:** 10.3389/fcvm.2023.1264398

**Published:** 2023-09-08

**Authors:** Guido Iaccarino

**Affiliations:** Director of the Interdepartmental Center for Hypertension and Related Conditions, Department of Clinical Medicine and Surgery, Federico II University of Naples, Naples, Italy

**Keywords:** innovation, hypertension, therapeutic, biomarkers, mechanisms of disease

**Editorial on the Research Topic** Insights in hypertension: 2022

## 1. Introduction

Is there a future for hypertension research and management, and what does it look like? This question was raised by Doctor Victor Dzau in an editorial published on Hypertension in 2019 (1). In that paper, he observed that the lack of novel therapeutic targets for hypertension drug development would have changed the objective of the research of the “Silent Killer” toward meaningful improvements in Hypertension Control and Management through a transformative system-wide, cross-sector approach, which should include digital, data science and artificial intelligence, biotech, biomedical, healthcare delivery, and population science transformation. Almost 5 years later, can we be appreciative of Victor Dzau's vision?

The Frontiers in Cardiovascular Medicine's Hypertension Research Topic dedicated to Insights in Hypertension 2022 seems to confirm new trends in hypertension management and contradict the issue that novel therapeutic targets are not in sight. Let us try to recapitulate what happened last year on the Insights.

## 2. Old and new biomarkers for high blood pressure and hypertension-related target organ damage

### 2.1. Gut Microbiota and betaine levels

The complex interplay between gut microbiota, its metabolites, and the development and evolution of cardiovascular diseases has been the objective of much recent research investigation. Metabolites from the choline pathway linking with the gut microbiota can increase in the blood and participate in defining the cardiovascular risk and the target organ damage. In particular, the products of the choline metabolism as gut microbiota–dependent have been considered for monitoring the effects of gut microbiota, particularly Betaine, which is the oxidized form of choline (2). These mechanisms are so promising that it is even possible to hypothesize possible interventions to modulate the gut microbiota to intervene in cardiovascular conditions or at least reduce cardiovascular risk (3). Li et al. exploit in their paper the link between the gut microbiome and hypertension, unveiling that altered gut microbiota is a causal factor in the development of hypertension. Much research is also needed to verify the reproducibility of these findings in different ethnicities and dietary habits, but it has posed the basis for investigating the possibility that diet interferes with hypertension by modulating the intestinal flora and the patterns of released metabolites. Interestingly, this mechanism might also influence the evolution of other conditions, such as pulmonary hypertension, as demonstrated by Yang et al. Indeed, they demonstrated that high plasma betaine levels are associated with pulmonary hypertension severity and it might participate to worsen the prognosis.

### 2.2. miRNA as a biomarker for primary hyperaldosteronisms

miRNAs are a class of non-coding small RNA molecules that regulate gene expression by a series of mechanisms, from gene expression repression/enhancement to mRNA stability and degradation. Subclasses of miRNA are more dedicated to the regulation of metabolism, such as insulin resistance or insulin sensitivity (4), and their regulation has been studied in several conditions but never in aldosterone-secreting adenomas. In their paper, Concistrè et al. show significantly reduced periadenoma fat tissue expression of miRNA-143 which is associated with a better clinical response to pharmacological and definitive surgical treatment. Thus, these miRNAs might represent a leading biomarker for the clinical follow-up of aldosterone-secreting adenomas.

## 3. New perspective on hypertension-related target organ damage

### 3.1. NAFLD and LV changes

The hypertension mechanism of disease includes insulin resistance, which among other things also participates in the development of hypertension-related target organ damage (5). It is therefore expected that the damage caused by insulin resistance in other organs (in this case the liver, target of Non-Alchoolic Fatty Liver Disease) would be somehow predictive of hypertension-related TOD (in this case Left Ventricle Hypertrophy and diastolic dysfunction). In this view, the association demonstrated by Professor Leonardo Sechi’s group between NAFLD and left ventricle hypertrophy is not surprising. What is surprising, though, is that this association appears to be independent of insulin sensitivity. In this case, the association would recognize a hemodynamic mechanism, which deserves further investigation.

### 3.2. Arterial stiffness and prothrombotic state

Arterial stiffness assessment in the clinical practice for the management of the hypertensive patient is an acquisition of the late eighties (6). It can be considered a maker of vascular TOD in hypertensives. It involves another relevant mechanism of disease which is endothelial dysfunction. It is interesting to note that the many functions of the endothelium also include the homeostasis of thrombosis and hemostasis (PMID: 23905034). It is interesting to note that in this issue, Brosolo et al. observe the association between arterial stiffness and prothrombotic state in uncomplicated hypertensives. This association, albeit anticipated, was never demonstrated and the authors filled a gap in knowledge with their paper.

### 3.3. Aging and hypertension in kidney function decline

Kidney function assessed by estimation of the Glomerular Filtration Rate is characterized by a physiological decline along aging (7). The speed of such a decline, though, is still an argument of debate. Some reports indicate that the speed at which kidney function declines is the same across all years though other reports suggest that the speed accelerates with age. It is clear that comorbidities, whose frequency increases with age, may participate in the process and therefore be confounders. In this scenario, the paper from Professor Ponte’s group sheds new light, demonstrating that the increase in the speed of kidney function decline is only observed in hypertensive patients.

## 4. Innovative therapeutic approaches

### 4.1. Therapeutic concordance for adverse drug-related events

Multimorbidity and polypharmacy are two sides of the same coin. Of course, they are intrinsically linked, but the unavoidability of complex therapeutic regimens is often the result of physician inertia, leading to adverse drug-related events. To avoid this phenomenon, different approaches such as medication review, decision support software, and drug interaction databases have been proposed (8). Trimarco et al. tested a solution including a trained pharmacy, whose primary responsibility was assessing the medical pharmacological history of poly-treated resistant hypertensives patients. Based on the potential interactions, the specialists suggested changes in therapy accordingly. Interestingly, this approach resulted in the reduction of the Adverse Drug-Related event rate from 70% to 25% in almost 3 years of follow-up. On the other end, though, there was the failure in obtaining a high rate of blood pressure control in treatment-resistant hypertensives.

### 4.2. Aprocitentan for resistant hypertension

Resistant hypertension is going to be the new frontier of hypertension. True resistant hypertensives are considered as those that do not reach blood pressure control even in the presence of heavy therapy including at least three drugs, including a diuretic at maximal tolerated dosage. Why are these patients not obtaining the expected success? This is often due to atypical hypertensive states that include disease mechanisms that are often not considered for a series of reasons, which include the lack of available therapeutic tools. Recently, aprocitentan, a non-selective endothelin Receptors Blocker, has been tested in a large clinical trial dedicated to resistant hypertensive patients. Varzideh et al. review the pros and cons of this study, whose name is “PRECISION” (9). The verdict is not out yet on whether this drug will be a major player in the future management of resistant hypertensives; nevertheless, its presence suggests that thinking outside the usual box when considering therapy for hypertensive patients might bring tailored therapies to different hypertensive phenotypes and more tailored therapeutics.

## 5. Conclusion

**Figure 1:**
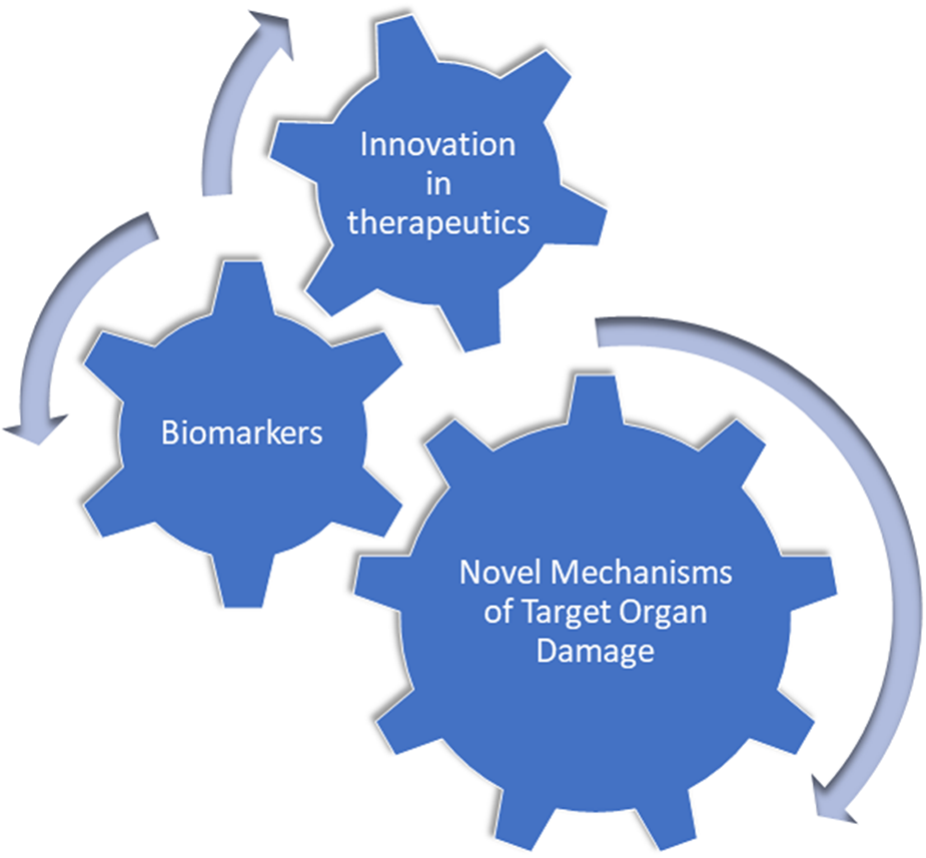
Insights in hypertension.

This issue of the Insights in Hypertension 2022 reveals the strict interconnection between the investigation of novel mechanisms of disease and the possibility of better characterizing with innovative biomarkers the phenotypes of our hypertensive patients to tailor innovative therapies to selected cases (Figure 1). Indeed, not all hypertensives are created equal, and different pathophysiology mechanisms can account for the same increase in the number of mmHg. Blood pressure measurement, on the basis of which we propose and monitor therapy, is probably a too gross way to stratify our hypertensive patients.
